# The Prevalence of p16 Expression in Urothelial Bladder Cancer in a Tertiary Care Hospital of Chattogram, Bangladesh

**DOI:** 10.30699/IJP.2023.1987551.3070

**Published:** 2023-07-16

**Authors:** Taniza Farnaz, Pradip Bhattacharjee, M. Shahab Uddin Ahamad, , Sharmin Ashraf Rima, Naznin Nahar Momin, Anika Sadaf

**Affiliations:** 1 Department of Pathology, Chittagong Medical College, Chattogram, Bangladesh; 2 Chattogram Ma O shishu Medical College, Chattogram, Bangladesh

**Keywords:** Grading, Immuno expression of p16, Muscle invasiveness, Urothelial carcinoma

## Abstract

**Background & Objective:**

p16 is a tumor suppressor gene, loss of which is usually associated with poor epithelial differentiation, resulting in tumor progression, which correlates with aggressive clinical behavior and poor prognosis. CDK 4/6 inhibitors can be used as a therapeutic target in p16 negative cases. Bladder cancer is one of the most prevalent cancers, prognosis of which depends not only upon the histopathological type, grade, and invasiveness but also on many other factors. The purpose of this study was to examine p16 expression in bladder urothelial carcinoma among the people who receive treatment at a tertiary care facility in Chattogram, Bangladesh.

**Methods:**

At the Department of Pathology, Chittagong Medical College we did this cross-sectional study from July 2019 to September 2021. The study included fifty-one cases of primary urothelial bladder cancer for histopathological examinations. Immunostaining was done by using a primary antibody against p16.

**Results:**

Among the 51 cases, twenty-six cases (51%) showed positive p16 expression. The proportion of patients with high-grade (66.7%) and muscle-invasive (86.4%) tumors were more prone to show p16 negativity.

**Conclusion:**

The result of this study shows the high grade and muscle-invasive urothelial bladder cancer is linked to reduction of p16 expression, which may provide additional prognostic information to stratify the high-risk patients and can also guide treatment plans, being a therapeutic target.

## Introduction

One of the most prevalent urogenital tract cancers is urinary bladder carcinoma (1). It is the world's ninth-most prevalent malignancy and the thirteenth-most frequent cause of cancer mortality (2). Previously urinary bladder cancer was common in developed countries, but in recent eras, its prevalence is increasing remarkably in developing countries, including Bangladesh due to many factors like changes in the environment, personal habits, occupation, and other factors (3-7). 

Muscle-invasive bladder cancer (MIBC) is a term used to describe tumors that invade the detrusor muscle, and they are more likely to spread to the lymph nodes and other organs (2). Patients with NMIBC who are not treated eventually develop MIBC, which has a far poorer prognosis (8-10). Grade, invasiveness, and lymph node metastasis are verified prognostic factors for urothelial carcinoma, but these are not adequate to determine which patients will have metastases or recurrence (11-13). 

The partial deletion of chromosome 9 is the genetic damage that occurs most frequently in urothelial cancer. One of the major loci for deletion is close to the CDKN2A/ARF gene on chromosome 9p21 (14). The CDKN2A gene produces the kinase inhibitor p16 (15). 

Numerous cancers, including urothelial malignancy, frequently have promotor hyper methylation, point mutations, and p16 deletion (11, 16). Many previous studies showed variations in the expression of p16 in urothelial cancer. Poor prognosis in bladder cancer is usually correlated with low expression of p16 (17). Aberrant expression of p16 can be regarded as a factor in urothelial carcinoma carcinogenesis and progression of tumor (16).

Expression of p16 can guide selection of therapeutic strategies for advanced bladder cancer (18). PD- 0332991 (Palbociclib) a selective CDK 4/6 inhibitor can be used as a therapeutic target in advanced bladder cancer for p16-negative cases by controlling cell cycle progression along with or in patients resistant to traditional therapies (18). Therefore, p16 is a crucial marker for patients with urothelial bladder cancer to predict recurrence, progression of the disease, and therapeutic target for treatment (11).

The present study is intended to observe the prevalence of p16 expression and its association with tumor grade and muscle invasiveness in urothelial carcinoma of the urinary bladder among the patients undergoing treatment at a tertiary care facility of Chattogram city in Bangladesh which may help to determine the prognosis of patients in our population and can open a door for planning newer therapeutic strategies for the patients who are resistant to traditional therapies.

## Material and Methods

This cross-sectional observational study was carried out from September 2019 to August 2021 at the Department of Pathology at the Chittagong Medical College in Chattogram, Bangladesh. Institutional Review Board (IRB) prior approval was obtained by the authors. Every possible case of urinary bladder cancer was investigated. Patients with primary urothelial carcinoma underwent transurethral resection of bladder tumor (TURBT) were selected as case but those who had undergone radiation or chemotherapy for a bladder tumor in the past were excluded. Patients were included finally if their case samples examined histologically and were then determined to have primary urothelial carcinoma by a minimum of two pathologists. To determine the sample size, the following formula is used:

$n=\frac{Z^{2}\mathrm{pq}}{d}$ here, p= Sample proportion or percentage of prevalence 3.4% (5).

Finally, 51 urothelial carcinoma cases were suitable for the immuno-histochemical analysis. Control for confounding was done by multivariate analysis. Data on variables of interest were recorded on a pre-designed record form.

According to WHO grading system tumor was divided into two groups high grade and low grade, Muscle invasiveness was considered for the invasion of detrusor muscle by malignant cells microscopically (2,19). 

Three-five μm thick sections of paraffin-embedded tissues were obtained for immunohistochemistry and placed on a slide coated with poly-L-lysine. Antigen retrieval was carried out, using a microwave at 750°C for 15 minutes after de-paraffinization and rehydration. After that, the sections had been stained for 30 minutes with a primary antibody. Invitrogen Polyclonal Mouse Anti-Human p16 antibody was used as the primary antibody. As a secondary antibody, DAKO REALTM EnVision was utilized. The sections were counterstained with Mayar's hematoxylin. Dehydration and mounting by DPX were done.

The extent of reactivity and the proportion of reactive cells were used to determine P16 status. Staining of the nucleus was considered positive. The proportion of reactive cells was scored from 0 to 4, and the intensity score was graded from 0 to 3. In order to arrive at the ultimate score, multiplied both, with a score range of 0 to 12. The final score (0-3) was considered negative and (4-12) was positive for p16 immunoexpression (20, 21).

**Statistical Analysis**

Data analysis was performed by using SPSS 25 (SPSS Inc., Chicago, Ill., USA). To determine any potential associations with various factors, the unpaired t-test, Chi-square test, and Fisher’s exact test were used. The threshold for statistical significance was a P-value of 0.05.

## Results

Male to female ratio in the 51 instances was 3.3:1, with 39 (76.5%) patients being male and 12 (23.5%) females. The patients ranged in age from 43 to 82, with the youngest being 43. The majority of the patients, (29.4.6%) were between 61 to 70 years of age. 

Patients' socioeconomic profiles were categorized using the modified Kuppuswamy Socioeconomic Status Scale (22). Thirty patients (58.8%) of the total from low and 21 patients (41.2%) were from middle socio-economic status. No case of the upper class was found in our study. Among them, 42 were non-industrial workers and others were industrial. 

Regarding personal habits 32 patients were smokers, and 24 had a habit of betel chewing. Eleven had a habit of both smoking and betel nut but 6 had none. No statistical significance was found with tumor grade and degree of muscle invasion with age, sex, socioeconomic status, or smoking habit but 79.2% of betel chewers had high-grade tumors. 

Out of 51 cases on the right or left lateral bladder wall, 19 of the tumors were discovered. Rests were performed on the base, numerous locations, the posterior wall, and the anterior wall, and in 05 (09.8%) cases, the site was not mentioned. Through a CT scan or ultrasonography, the area was evaluated.

Patients with no substantial prior history were the majority (41 instances; 80.4%) of cases. Seven patients (13.7%) had a history of chronic bladder inflammation, and two patients (03.9%) took immunosuppressive medications for arthritis, 01 patient (02%) had a history of instrumentation for TURP. Among the 51 cases, 22 were hypertensive and 15 were diabetic and 08 had both.

Most of the patients 72.5% (n=37), presented with hematuria, others had dysuria, lower abdominal pain, whole abdominal pain, and nonspecific symptoms.

On microscopic examination, among 51 cases 30 high-grade (58.8%) and 26 muscle-invasive (51%) cases were observed. Twenty-three of the 30 high-grade cases were MIBC and seven were NMIBC. A statistically significant difference between muscle invasiveness and different histological grades came to light. (*P*<0.001). Twenty-six cases (51%) showed p16 positivity and the rest were negative.

In age distribution out of the 51 patients, p16 expression was found to be negative in 14 (56%) of the patients and positive in 11 (44%) of the patients. in the ≤ 60 years age group. In >60 years age group, 15 (57.5%) cases showed negative expression, and 11 (42.3%) cases showed positive expression of p16. No significant relationship was also found with p16 expression and other non-clinical variables. 

But tumor grade and muscle invasiveness showed a remarkable relationship with p16 expression. The proportion of p16 negativity was significantly higher in patients with invasive, high-grade muscle tumors. Among 30 cases of high-grade tumor 10 (33.3%) and 20 (66.7%) cases showed p16 positivity and negativity, respectively. In the case of muscle invasiveness, among the twenty-six cases of MIBC, twenty-two cases (86.4%) showed negative expression of p16, and 04 (15.4%) cases were p16 positive. Both of them showed high statistical significance (*P*<0.05). Microscopic findings are discussed in Table 1.

Among the 07 high-grade NMIBC cases, 04 cases showed p16 positivity and 03 were negative and all the 03 low-grade MIBC were p16 negative which indicates the prognostic value of p16 in urothelial carcinoma.

Table 2 illustrates the frequency of p16 expression in bladder urothelial cancer in various studies.

**Table 1:** Distribution of the patients according to grade and muscle invasiveness by p16 expression (n=51)

	p16 Expression		**P-**value *
	Positive	Negative	
Grade Low, n=21	16 (76.2)	05 (23.8)	0.004 ^s^
High, n=30	10 (33.3)	20 (66.7)	
Invasiveness MIBC, n=26	04 (15.4)	22 (84.6)	<0.001 ^s^
NMIBC, n=25	22 (88.0)	03 (12.0)	

*Fisher’s exact test was done to measure the level of significance.

The figure within parenthesis indicates in percentage.

s=Statistically significant.

MIBC- Muscle Invasive Bladder Carcinoma

NMIBC- Non-Muscle Invasive Bladder Carcinoma

**Table 2:** Comparison of p16 expression in the urothelial carcinoma among different reported studies

Author	Country	Total number of cases	P16 expression Positive Negative	
Yang *et al*. (2014)	Taiwan	78	49%	51%
Lee* et al*. (2010)	Korea	103	35.9%	64.1%
Hashmi *et al.* (2019)	Pakistan	121	14%	86%
Nakazawa *et al*. (2009)	Japan	190	50%	50%
Rasopollini *et al*. (2006)	Italy	33	28.2%	71.80%
Alshaikhly *et al*. (2017)	Iraq	48	70.8%	29.2%
The present study (2021)	Bangladesh	51	51%	49%

## Discussion

In the present study, we observed 58.8% (n=30) of high-grade urothelial carcinoma, which showed predominance. According to Haque *et al*., (23) in Bangladesh, Chinnasamy *et al*., (1) and Chou *et al*. (24) found 72.0%, 63.4%, and 56.8% cases of high-grade tumors, respectively, which also showed a predominance of high-grade cases.

In the case of muscle invasiveness present study found 51% MIBC cases, whether Sadaf* et al.,* (24) recorded 71.43% MIBC cases, Chou *et al*., (24) found 59.5% MIBC cases showing a higher incidence of MIBC cases like the present study. The current investigation discovered that 76.7% of high-grade urothelial cancer patients were MIBC. Thapa *et al.,* (2017) reported that 24.45% of cases of high-grade tumors were muscle invasive (26).

According to Table 1, 20 (66.7%) of the thirty high-grade cancer patients revealed negative expression and 10 (33.3%) cases showed positive expression. Patients with low-grade malignancy had significantly higher expression of p16. This shows that the rate of loss of p16 is substantially increased (*P*=0.05) with the advancement in tumor grade, comparable to Yang *et al.* (2004) and Alshaikhly *et al.* (2017) who showed loss of p16 expression with increasing tumor grade in urothelial carcinoma but Hashmi *et al.* (2019) found 59.6% cases of low-grade urothelial cancer had been identified negative expression which does not correspond with the present study (11, 28, 29).

The results of this study, which examined the relationship between p16 positivity and the muscle-invasiveness of urinary bladder cancers, showed that 22 (88.0%) of the 25 NMIBC cases tested positive for p16. Whereas 26 MIBC cases showed 22 (84.6%) negative expression of p16. Differences between p16 expressions and muscle-invasiveness were obvious and statistically significant (*P*=0.05). According to Yang *et al.,* (2014), p16 expression was considerably greater in NMIBC compared to MIBC, and Alshaikhly *et al.,* (2017) similarly found that NMIBC patients with p16-positive tumors were more, with 26 out of 31 instances (11, 29). Hashmi *et al*. (2019) observed that MIBC considerably had a greater rate of p16 positivity (28). Variations in the procedure of tissue processing (specimen handling, fixation time, fixatives, paraffin block preparation, reagent quality, etc.) and immunohistochemistry technique (antigen retrieval, antibody dilution, incubation period, washing, etc.) among different researchers are usually liable for variations in p16 expression between studies.

The mean age of the patients in the present study was 62.41±5.2 years with male predominance. Male to female ratio was found 3.3:1. Kumar *et al.,* (2017) found Male to female ratio of 4:1 among patients with neoplastic lesions, with a mean age of 55.6 years (30). Gupta *et al.,* (2009) found the mean age of patients with bladder carcinoma was 60.2 ± 4.4 years with a male-to-female ratio of 8.6:1, which was higher than the present study (31). According to Rushton *et al.,* (2010), 5.6% of urinary bladder cancers in their study were linked to occupational exposure, which may be the reason why men are more likely to develop the condition (32).”

In the group of patients under 60 years old, 14 (56%) showed negative expression of p16. Fifteen (57.7%) of the patients in the over-60 age group displayed positive p16 expression. Out of the 39 male patients, 20 (51.3%) cases showed positive expression, and 19 (48.7%) cases showed negative expression of p16. No significant difference in the expression of p16 was found among different age groups or sex (*P*>0.05). No statistically significant correlations between p16 expression and various age and sex groups were discovered in the study by Yang *et al.,* (2014) and Hashmi *et al.,* (2019) (11,29).

## Limitations and Recommendations

All the patients in this study were operated on between 2019 and 2021 and as follow-up was not performed, there was no scope to study the prognosis of these cases with the result of the present study. Due to a lack of cystectomy specimens, data on metastases that could have provided a more solid assessment of the prognostic relevance of p16 was not able to be included.

Further studies with larger sample sizes, cystectomy specimens, more logistic supports, and proper follow-up should be carried out. A confirmatory test with FISH or RT-PCR for more reliable information can be carried out.

## Conclusion

In this study, it was observed that patients with high-grade and muscle-invasive urinary bladder urothelial carcinoma had a larger percentage, which showed p16 negativity. Based on the finding of the study, for identifying patients at risk, the expression profile of p16 may be helpful and can also guide the treatment plan being a therapeutic target for p16 negative cases, who can be treated by selective CDK 4/6 inhibitor in our population.

## Funding

None.

## Conflict of Interest

None.
